# 950 Demographic and Clinical Predictors of Suspected Abuse in Genital Burn Injuries: A National Database Study

**DOI:** 10.1093/jbcr/iraf019.481

**Published:** 2025-04-01

**Authors:** Hilary Liu, Mare Kaulakis, Christopher Fedor, José Arellano, Rebecca Hohsfield, Paul Rusilko, Garth Elias, Alain Corcos, Jenny Ziembicki, Francesco Egro

**Affiliations:** University of Pittsburgh Medical Center; University of Pittsburgh School of Medicine; University of Pittsburgh School of Medicine; University of Pittsburgh Medical Center; University of Pittsburgh Medical Center; University of Pittsburgh Medical Center; University of Pittsburgh Medical Center; University of Pittsburgh Medical Center; University of Pittsburgh Medical Center; University of Pittsburgh Medical Center

## Abstract

**Introduction:**

Genital burn injuries can have serious consequences and may raise concerns about potential abuse. Understanding the relationship between genital burns and suspected abuse is critical for improving patient care and developing evidence-based strategies. This study characterizes genital burn patients with suspected abuse and identify associated risk factors using a national database.

**Methods:**

A retrospective review was conducted using the ABA Burn Care Quality Platform (BCQP) data from January 2013 to December 2022 on genital burn patients suspected of abuse. Patient demographics, burn characteristics, and clinical data were analyzed. Multiple linear regression was used to evaluate factors associated with suspected abuse.

**Results:**

Of 11,222 patients (31.6% female, 68.4% male; mean age 33.2 ± 25.7 years) with genital burns, 782 patients (7.0%) were suspected of abuse. Males were slightly less likely to be suspected of abuse than females (OR = 0.989, p=0.033). Younger age was significantly associated with higher suspicion, with each year increase in age reducing the likelihood of suspected abuse (OR = 0.998, p< 0.001). Asian (OR = 0.919, p=0.002) and White patients (OR = 0.942, p=0.014) had lower rates of suspected abuse compared to other racial groups. Drug use (p=0.359) and marital status (p=0.848) were not significantly associated with suspected abuse. Patients living in houses/apartments (OR = 0.854, p< 0.001), institutions/prisons (OR = 0.886, p=0.022), and school dormitories (OR = 0.802, p=0.001) had lower rates of suspected abuse compared to those living alone.

In regard to burn characteristics, a larger total body surface area (TBSA) was associated with increased suspicion of abuse (OR = 1.0005, p< 0.001). Electrical burns (OR = 0.934, p=0.002), flame burns (OR = 0.971, p=0.027), and flash burns (OR = 0.951, p=0.004) were associated with lower rates of suspected abuse compared to other burn types.

**Conclusions:**

Several demographic and clinical factors, including younger age, female gender, larger TBSA burns, and living alone, are associated with higher rates of suspected abuse in genital burn patients. Drug use and marital status were not significant factors.

**Applicability of Research to Practice:**

These findings highlight the need for more tailored screening approaches for vulnerable patient subgroups, using identified risk factors to improve the detection and management of suspected abuse in genital burn cases.

**Funding for the Study:**

N/A

